# High expression of MnSOD promotes survival of circulating breast cancer cells and increases their resistance to doxorubicin

**DOI:** 10.18632/oncotarget.10360

**Published:** 2016-07-01

**Authors:** Afu Fu, Shijun Ma, Na Wei, Blanche Xiao Xuan Tan, Ern Yu Tan, Kathy Qian Luo

**Affiliations:** ^1^ School of Chemical and Biomedical Engineering, Nanyang Technological University, Singapore; ^2^ Department of General Surgery, Tan Tock Seng Hospital, Singapore; ^3^ Faculty of Health Sciences, University of Macau, Taipa, Macau, China

**Keywords:** MnSOD, breast cancer metastasis, circulating tumor cells, doxorubicin resistance, apoptosis

## Abstract

Understanding the survival mechanism of metastatic cancer cells in circulation will provide new perspectives on metastasis prevention and also shed new light on metastasis-derived drug resistance. In this study, we made it feasible to detect apoptosis of circulating tumor cells (CTCs) in real-time by integrating a fluorescence resonance energy transfer (FRET)-based caspase sensor into one *in vitro* microfluidic circulatory system, and two *in vivo* models: zebrafish circulation and mouse lung metastatic model. Our study demonstrated that fluid shear stresses triggered apoptosis of breast cancer cells in circulation by elevating the mitochondrial production of the primary free radical, superoxide anion. Cancer cells with high levels of manganese superoxide dismutase (MnSOD) exhibited stronger resistance to shear force-induced apoptosis and formed more lung metastases in mice. These metastasized cells further displayed higher resistance to chemotherapeutic agent doxorubicin, which also generates superoxide in mitochondria. Specific siRNA-mediated MnSOD knockdown reversed all three phenotypes. Our findings therefore suggest that MnSOD plays an important integrative role in supporting cancer cell survival in circulation, metastasis, and doxorubicin resistance. MnSOD can serve as a new biomarker for identifying metastatic CTCs and a novel therapeutic target for inhibiting metastasis and destroying doxorubicin-resistant breast cancer cells.

## INTRODUCTION

Most cancer patients, especially breast cancer patients, die of metastasis because metastatic tumors are difficult to remove surgically and often develop resistance to conventional chemotherapy [1]. In this study, we aimed to determine whether there is a common mechanism supporting both metastasis and drug resistance. We focused on circulating tumor cells (CTCs), as they are the “seeds” that potentially form metastatic tumors. Cancer cells can become CTCs by either actively invading nearby lymphatic vessels and capillaries or passively shedding into the bloodstream via leaky blood vessels formed during angiogenesis or surgery. Once tumor cells enter the bloodstream, most of them are eliminated in the circulation through several mechanisms: 1) anoikis due to the detachment of cancer cells from the extracellular matrix and the disruption of cytoskeleton, which lead to cell rounding [2]; 2) immune system-mediated destruction, which is executed by natural killer cells [3]; and 3) hemodynamic shear stress (SS), which is mainly generated from the blood flow but also from collisions between CTCs, blood cells, and endothelial cells lining the vessel wall. Among these three mechanisms, the most important one is the effect of fluid SS on the viability of CTCs because the greatest proportion of CTCs are destroyed by this mechanical shear force. Previous studies have shown that cancer cells could be killed in the circulation via deformation [4–8]. However, the questions of how fluid SS kills CTCs and how surviving CTCs resist shear force remain unanswered [9]. Understanding these issues will provide new insights on how CTCs survive SS to establish distant metastatic colonies.

Metastatic tumors are usually resistant to various cancer therapies [1]. Current breast cancer treatment guidelines recommend adjuvant systemic therapy following surgical resection of the primary tumor to treat CTCs and micrometastasis. Anthracycline-based chemotherapy regimens are among the recommended first-line regimens; among them, doxorubicin (DOX) is one of the commonly used drugs. In spite of such treatments, distant disease recurrence often occurs. This has been postulated to be a consequence of tumor resistance to conventional treatments – a hypothesis further supported by observations that metastatic disease often responds less effectively to treatment and is associated with poor prognoses. There has been extensive research into the mechanisms contributing to DOX resistance, including the over-expression of P-glycoprotein, multidrug resistance-associated proteins [10], and anti-apoptotic proteins Bcl-2 and Bcl-xL [11–13]. However, the reason why the response rate to DOX in metastatic cancers is only half of those observed in the primary tumors remains unknown [14, 15]. We therefore hypothesized that the CTCs that survived the fluid SS in the bloodstream may possess some special properties, which help them to become metastatic and resistant to DOX.

To find connections between cell survival, metastasis and DOX resistance, we generated breast cancer cells with different metastatic potentials and engineered those cells to produce a fluorescence resonance energy transfer (FRET)-based caspase sensor (sensor C3) [16] for the real-time detection of apoptosis. We also generated three model systems, including a microfluidic circulatory system for generating pulsatile fluidic flow, a zebrafish tumor model, and a mouse lung metastasis model. By integrating the sensor cells into these three model systems, we identified an antioxidant enzyme, MnSOD, as a common foundation supporting cancer cell survival in the circulation and metastasis, and increasing cell resistance to DOX.

## RESULTS

### Metastatic breast cancer cells survive in mice circulation by resisting apoptosis

**Figure 1:**
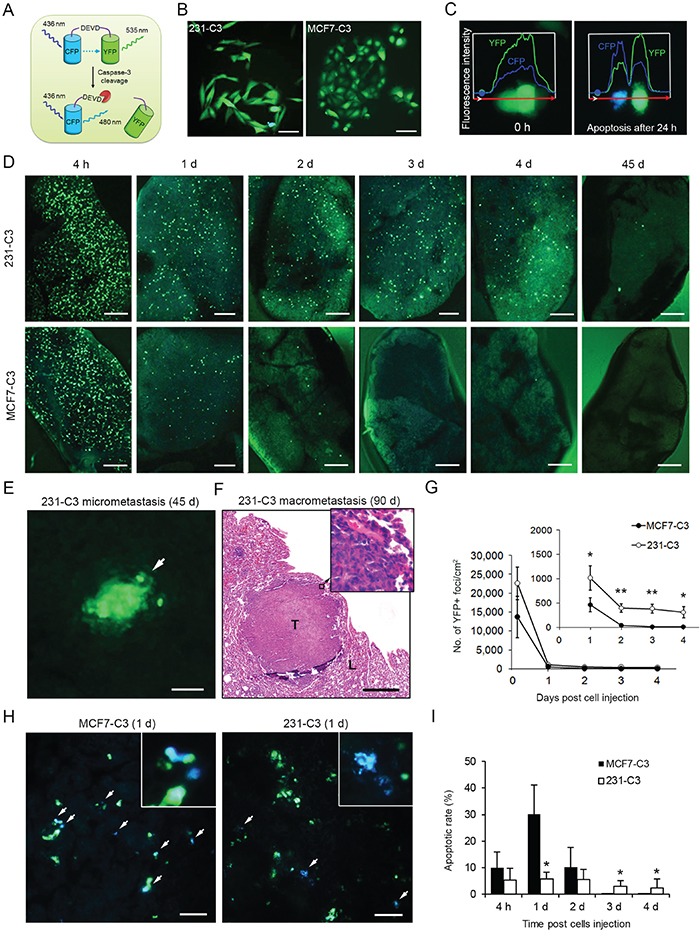
231-C3 cells are more metastatic and durable than MCF7-C3 cells in mice **A.** Schematic representation of FRET sensor for detecting caspase-3/−7 activation during apoptosis. **B.** FRET images of 231-C3 and MCF7-C3 cells in normal condition. Scale bars represent 100 μm. **C.** Fluorescence intensities of YFP and CFP of MCF7-C3 cells at 0 and 24 hours after 3 minutes of UV irradiation. **D, G-I.** Metastatic ability was determined by injecting sensor cells into the tail vein of nude mice and observing metastases on the lung. Distribution of 231-C3 and MCF7-C3 cells on the lung was revealed by FRET imaging (D, scale bars represent 1 mm). Cell viability was determined by counting YFP+ cells from 0-4 days (outer panel) and 1-4 days (inner panel) (G). Apoptotic rates (I) were determined by analyzing the FRET images (H, white arrows indicate blue apoptotic cells). Scale bars represent 100 μm. **E** and **F.** Representative FRET image of 231-C3 micrometastasis (white arrow) at day 45 (E), and H&E staining of a macrometastatic tumor (T) in lung tissue (L) at day 90 (F). Scale bar represents 100 μm in (E) and 500 μm in (F). The data are the mean ± SD from all lung samples of all three mice. ^*^P < 0.05, ^**^P < 0.01 by Student's *t* test, 231-C3 *vs*. MCF7-C3 cells.

We previously constructed a caspase sensor for the real-time detection of caspase-3/−7 activation during apoptosis (Figure 1A) [16]. Recently, we generated two stable sensor 231-C3 and MCF7-C3 cell lines from metastatic breast cancer MDA-MB-231 and non-metastatic breast cancer MCF7 cells (Figure 1B) [17]. These sensor cells could change their color from green to blue under UV-irradiation-induced apoptosis due to the reduction of fluorescence resonance energy transfer (FRET) between the donor, cyan fluorescent protein (CFP), and the acceptor, yellow fluorescent protein (YFP) (Figure 1A-1C and Supplementary Video S1). In this study, a cell was defined apoptotic if its FRET effect, which is the fluorescence emission ratio of YFP to CFP, was reduced by ≥ 50% (Supplementary Figure S1A and S1B).

To compare the metastatic ability of the 231-C3 and MCF7-C3 cells, a lung metastasis experiment was conducted in mice. Cells of equal numbers (5 × 10^5^) were injected into the tail vein of nude mice. The number of sensor cells in the lung was counted while no sensor cells were detected in other parts of the animal 24 hours after the injection. The 231-C3 cells displayed much higher viability than MCF7-C3 cells in lung tissue (300-400 cells/cm^2^ vs. almost none) three days after injection (Figure 1D and 1G). Some 231-C3 cells eventually formed micrometastases at 45 days (Figure 1D and 1E) and macrometastases at 90 days (Figure 1F).

To understand why the 231-C3 cells were more viable than the MCF7-C3 cells in mice, we conducted a more-detailed, *in situ* single-cell apoptosis analysis on the sensor cells found in the lung. The FRET imaging analysis showed that the apoptotic rate of the 231-C3 cells was five times lower than the rate of the MCF7-C3 cells (5.8 ± 2.6% vs. 30.2 ± 11.0%) (Figure 1H and 1I). Together, these results show that 231-C3 cells are more metastatic and durable than MCF7-C3 cells; the results also imply that most injected sensor cells died during the circulation.

### Metastatic cells are more resistant to hemodynamic SS-induced apoptosis in zebrafish

**Figure 2:**
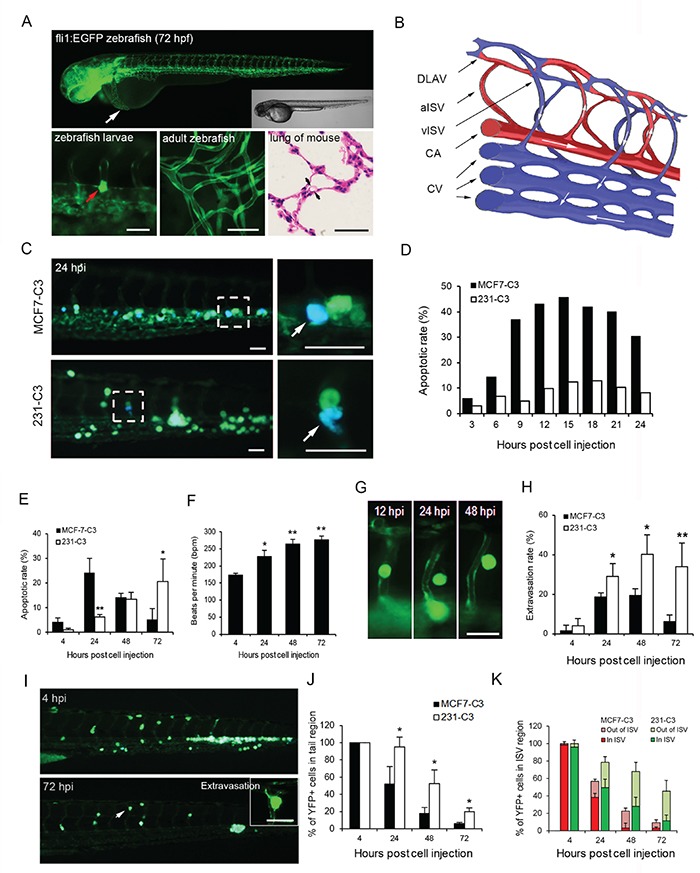
Metastatic cells are more resistant to SS-induced apoptosis in zebrafish **A.** Upper panel: transgenic *Tg (fli1:EGFP)* zebrafish larvae expressing EGFP in the vascular system at 72 hours post fertilization were visualized using fluorescence and DIC microscopy. The white arrow indicates the injection site of cancer cells. Lower panels: larval zebrafish blood vessel diameter (left) in comparison with those of adult zebrafish capillaries (middle) and mouse pulmonary alveoli (right). A cancer cell larger than the small blood vessel is indicated by a red arrow (left). **B.** Schematic diagram illustrating the structure of blood vessels of zebrafish in the observation window. DLAV: dorsal longitudinal anastomotic vessel, aISV: arterial intersegmental vessel, vISV: venous intersegmental vessel, CA: caudal artery, and CV: caudal vein. **C-E.** The apoptotic rates of sensor cells circulating in zebrafish were determined by FRET imaging analysis. Representative FRET images of sensor cells with a blue apoptotic cell enclosed in the dashed boxes and enlarged in the right panels (C). Quantified apoptotic rates within 24 (D) and 72 hours post injection (E); *n* = 200-300 cells at each time point. **F.** Heart rates in control zebrafish larvae were counted after cells were injected. **G** and **H.** Extravasation of sensor cells was determined by their position in ISVs of the tail region by YFP imaging. YFP images of MCF7-C3 cells during extravasation (G) and rates of sensor cell extravasation (H). **I-K.** Location of 231-C3 cells in the tail region of zebrafish revealed by FRET imaging (I). Percentages of YFP+ sensor cells located in the whole tail region (J), or cells located in and outside of the ISVs (K) were determined by counting cells; *n* ≥ 5 fish, and *n* = 20-50 sensor cells per fish. The data are the mean ± SD. ^*^P < 0.05, ^**^P < 0.01 by Student's *t* test: 231-C3 *vs*. MCF7-C3 cells. All scale bars represent 50 μm.

To investigate how cancer cells were eliminated in the circulation, we used 3-6 day-old larvae of a transgenic zebrafish line, *Tg (fli1:EGFP)*, which expresses EGFP in its vascular system (Figure 2A). Despite its young age, zebrafish larvae already have a pulsatile blood flow (Supplementary Video S2). As zebrafish larvae have transparent bodies, sensor cells can be clearly identified in different parts of the blood vessels, such as the caudal artery (CA), caudal vein (CV), and intersegmental vessels (ISVs) (Figure 2B). ISV diameter is similar to that of capillaries in adult zebrafish or pulmonary alveoli in mice, ranging from 5-10 μm, which is smaller than the average size of a CTC (15-20 μm); CTCs can thus be arrested by ISVs (Figure 2A, red arrow).

Both two types of sensor cells (50-100 cells) were injected into the pericardium (Figure 2A, white arrow) of a larva at 72 hours post fertilization, and approximately 20-50 cells entered the circulation and moved to the tail region. These sensor cells were observed to circulate in large vessels such as the CA and CV, subsequently trapped in small vessels such as ISVs and CV plexus, or returned to circulation via moving through small vessels (Supplementary Figure S2A). More strikingly, most MCF7-C3 cells underwent apoptosis in the blood stream of zebrafish within the first 24 hours post injection, while most 231-C3 cells survived until 72 hours post injection (Figure 2C-2E). These results show that non-metastatic MCF7-C3 cells died much faster through apoptosis in zebrafish circulation than metastatic 231-C3 cells.

Several pieces of evidence suggested that the injected sensor cells might encounter destructive shear forces in zebrafish circulation. First, the sensor cells experienced pulsatile SS in the circulation (Supplementary Video S2). Second, the rate of 231-C3 cell apoptosis increased along with the elevation of zebrafish heartbeats during the development (Figure 2E and 2F). Third, more 231-C3 cells, compared to MCF7-C3 cells were found to extravasate (Figure 2G and 2H) or move from large vessels to ISVs at the tail region (Figure 2I and 2J, Supplementary Figure S2B and S2C) and the survival rates of 231-C3 cells located outside of ISVs at 48-72 hours post injection were much higher than those of cells that remained inside (Figure 2K). Finally, no viable cells were found within large vessels, such as the CA and CV, at 72 hours post injection unless they had formed a cluster, which might shelter them from the higher fluid shear forces in large vessels (Supplementary Figure S2B and S2D).

### Fluid SS triggers cancer cell apoptosis in a microfluidic circulatory system

**Figure 3:**
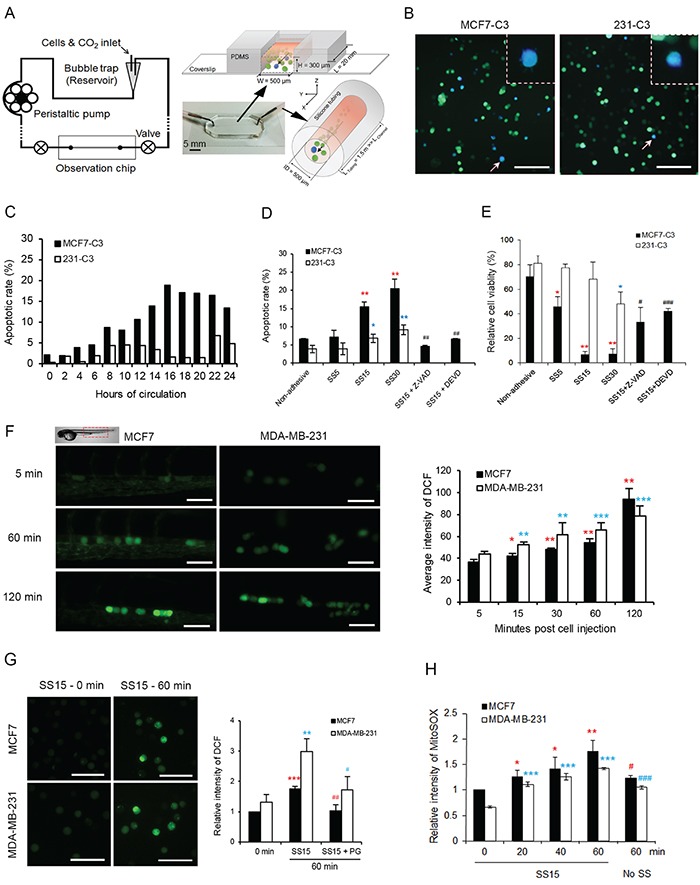
Fluid SS elevated levels of ROS and induced apoptosis in CTCs **A.** Diagram of a new microfluidic circulatory system for generating pulsatile SS. **B** and **C.** Effect of SS15 on apoptosis was determined in sensor cells. Representative FRET images of sensor cells with apoptotic blue cells indicated by white arrows and enlarged in the dashed boxes (B). Scale bars represent 100 μm. Apoptotic rates were determined by FRET imaging (C); *n* > 200 sensor cells for each time point. **D** and **E.** Apoptotic rates were determined by FRET imaging (D), and cell viabilities were quantified by the MTT assay (E) in sensor cells pre-treated with or without Z-VAD-FMK (Z-VAD, 20 μM) or caspase-3/−7 inhibitor Ac-DEVD-CHO (DEVD, 10 μM) for 1 hour. Cells grown in non-adhesive-coated wells were used as a negative control. ^*^P < 0.05, ^**^P < 0.01 by Student's *t* test: SS5-SS30 vs. non-adhesive condition. ^#^*P* < 0.05, ^##^*P* < 0.01, ^###^*P* < 0.001 comparing with and without inhibitors under SS15 treatment. **F.** ROS levels were determined by CM-H_2_DCFDA staining-based fluorescence microscopy in MCF7 and MDA-MB-231 cells injected in zebrafish larvae. *n* = 100-200 cells from > 10 fish. Scale bars represent 50 μm. **G.** ROS levels were measured as described in (F) from cells that circulated under SS15 in a microfluidic system with or without 20 μM PG. The average intensity from 200 cells was calculated in each sample, and the results represent the mean ± SD from three independent experiments. ^**^P < 0.01 and ^***^P < 0.001 by Student's t test: 60 vs. 0 minute.^#^*P* < 0.05, ^##^*P* < 0.01, comparing with and without PG under SS15 for 60 minutes **H.** Levels of mitochondrial superoxide were determined by MitoSOX (10 μM) staining and flow cytometry analysis. A non-adhesive condition with no shear stress was used as a negative control. The average intensity from 10,000 cells was calculated in each sample, and the results represent the mean ± SD from three independent experiments. ^*^P < 0.05, ^**^P < 0.01 and ^***^P < 0.001 by Student's t test: other times vs. 0 minute.^#^*P* < 0.05, ^##^*P* < 0.01, ^###^*P* < 0.001 comparing SS15 with no shear at 60 minutes.

To explore the impact of fluid SS on circulating cells, we designed a microfluidic circulatory system that uses a peristaltic pump to generate pulsatile shear forces similar to those produced in human vasculature (Figure 3A, Supplementary Video S3). Three levels of SS, 5, 15 and 30 dyne/cm^2^, were generated using this system, which represent venous SS (0.5-4.0 dyne/cm^2^) and arterial SS (4.0-30.0 dyne/cm^2^) in human circulation [18]. Sensor cells were circulated in this system for different durations, and their apoptotic status was determined using FRET imaging through a PDMS-based observation chip (Figure 3A and 3B). Circulatory treatment under SS15 resulted in a 4-fold higher apoptotic rate in MCF7-C3 than in 231-C3 cells (18.9% vs. 4.6%) within 20 hours of circulation (Figure 3C). A significant increase in apoptotic rates were observed in MCF7-C3 cells compared with that in 231-C3 cells when the SS levels were elevated (Figure 3D and 3E). More importantly, inhibiting caspase activation using a pan-caspase inhibitor (Ac-DEVD-CHO) or a caspase-3/−7 inhibitor (Z-VAD-FMK) significantly rescued SS-induced cell death (Figure 3D and 3E), indicating that apoptosis is the major type of cell death caused by fluid SS.

### Fluid SS increases the levels of mitochondrial superoxide in CTCs

In our previous studies, we showed that fluid SS induced endothelial cell apoptosis by elevating reactive oxygen species (ROS) levels [19, 20]. To determine whether fluid SS has similar effects on CTCs, we measured the levels of ROS by pre-incubating cells with an ROS-detecting dye, CM-H_2_DCFDA (10 μM), for 30 minutes before injecting them into either zebrafish circulation or a microfluidic system. Because this ROS dye can emit green fluorescence, we used non-fluorescent, parental cell lines of sensor cells, MCF7 and MDA-MB-231, in this experiment. Significant increases of ROS level were detected in both cell types 60-120 minutes after being injected into zebrafish (Figure 3F) or after 60 minutes of circulating in the microfluidic system under SS15 (Figure 3G, Supplementary Figure S3A-S3C). Pre-treating cells with 20 μM propyl gallate (PG) for 60 minutes almost completely prevented ROS elevation (Figure 3G). Because PG can scavenge superoxide and hydroxyl radicals [21, 22], we then used a mitochondrial superoxide-specific dye, MitoSOX Red, to show that fluid SS elevated the levels of mitochondrial superoxide in both MCF7 and MDA-MB-231 cells, regardless of their metastatic abilities (Figure 3H and Supplementary Figure S3D).

### SS causes more mitochondrial damage and cell death in non-metastatic cells

**Figure 4:**
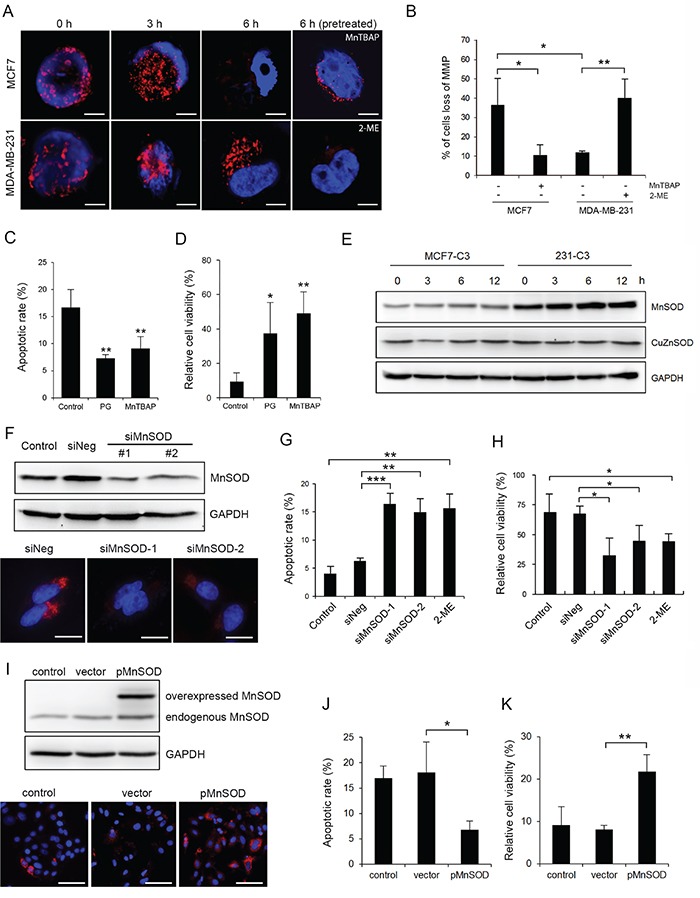
MnSOD protects metastatic cells from SS-induced mitochondrial damage and apoptosis **A** and **B.** Mitochondrial membrane potential (MMP) was examined by staining MCF7 and MDA-MB-231 cells with MitoTracker Red-CMXRos after circulation. MCF7 cells were pre-treated with MnTBAP (100 μM) and 231 cells were pre-treated with 2-ME (20 μM) before circulation (A). Scale bars represent 5 μm. The percentages of cells with lost MMP were quantified from more than 200 cells (B). **C** and **D.** MCF7-C3 cells were pre-treated with or without PG (20 μM) or MnTBAP (100 μM) and circulated for 18 hours. Apoptotic rates were measured by FRET imaging (C), and cell viabilities were quantified using the MTT assay (D). **E.** Expression of CuZnSOD and MnSOD was determined by Western blotting. GAPDH was used as a loading control. **F.** Knockdown efficiency of MnSOD in 231-C3 cells was examined by Western blotting and IF staining. Scale bars represent 10 μm. **G** and **H.** Effects of MnSOD knockdown using siRNAs or inhibiting its activity using 20 μM 2-ME on 18 hours of SS-induced apoptosis were determined by FRET imaging in 231-C3 cells (G), and those same effects on cell viability were measured using the MTT assay (H). **I-K.** Effects of overexpression of MnSOD on 18 hours of SS-induced apoptosis were determined. Expression of MnSOD in MCF7-C3 cells transfected with MnSOD plasmid or vector alone was examined by Western blotting and IF staining (I). Apoptotic rates were determined by FRET imaging (J), and cell viability was measured using the MTT assay (K). The results represent the mean ± SD from three independent experiments. ^*^P < 0.05, ^**^P < 0.01, ^***^P < 0.001 using Student's *t* test either comparing experimental groups with the control group or as indicated in the graphs. All cells subjected to the microfluidic circulatory system in this figure were under SS15. Nuclei were stained with Hoechst 33342 (blue). Scale bars represent 100 μm.

**Figure 5:**
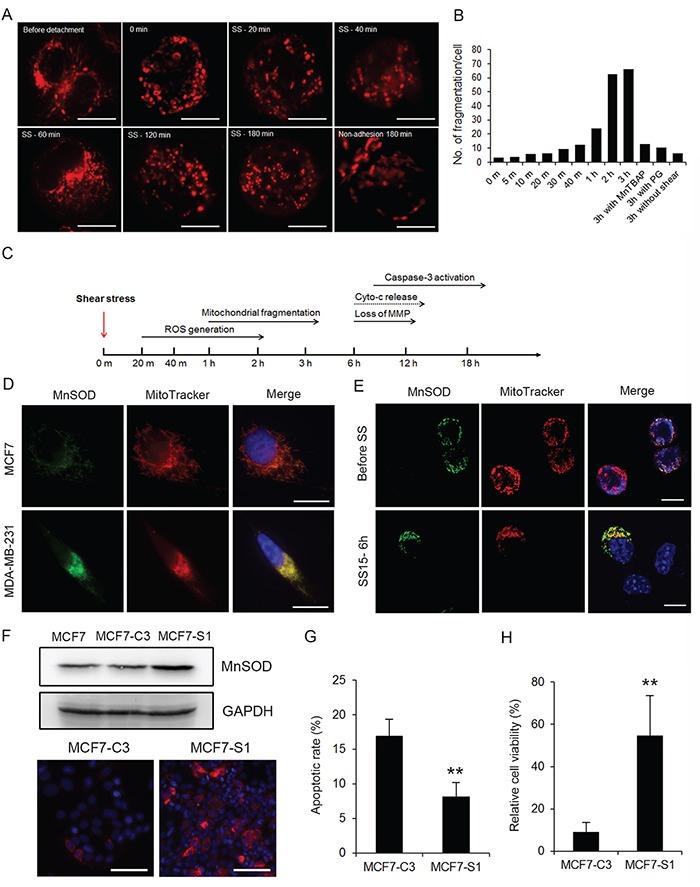
High levels of MnSOD protect cancer cells from fluid SS-induced oxidative stress and mitochondrial damage **A.** Real-time imaging of fluid SS caused mitochondrial fragmentation in MCF7 cells after 0, 20, 40, 60, 120 and 180 minutes of circulation. Mitochondrial morphology was monitored by pre-staining cells with 100 nM MitoTracker Red. Cells grown in a non-adhesive dish were used as a negative control. Scale bars represent 10 μm. **B.** The fragmentation of mitochondria in circulating MCF7 cells was quantified from 10-15 cells in each condition. A mitochondrion was considered fragmented if its diameter was less than the average diameter of mitochondria in attached cells. The average number of fragmentations was calculated and presented in the bar graph. **C.** Time line of SS-induced effects on ROS generation, mitochondrial damage and induction of apoptosis. **D.** Co-localization of MnSOD (green) and MitoTracker (red) in MCF7 and MDA-MB-231 cells. Nuclei were labeled with Hoechst 33342 (blue). Scale bars represent 10 μm. **E.** Expression of MnSOD (green) was detected in a few MCF7 cells, which protected these cells from mitochondrial damage, as indicated by MitoTracker staining (red). Nuclei were labeled with Hoechst 33342 (blue). Scale bars represent 10 μm. **F.** The MCF7-C3 cells survived after circulation for 18 hours were cultured into a new cell line, MCF7-S1. The expression levels of MnSOD in MCF7, MCF7-C3 and MCF7-S1 cells were determined by Western blotting and immunostaining (red). Nuclei were labeled with Hoechst 33342 (blue). Scale bars represent 100 μm. **G** and **H.** MCF7-C3 and MCF7-S1 cells were circulated for 18 hours. Their apoptotic rates were quantified using FRET imaging (G), and cell viability was measured using the MTT assay (H). The results represent the mean ± SD from three independent experiments. ^**^P < 0.01 using Student's *t* test comparing with MCF7-C3 in the graphs. All cells represented in this figure were subjected to SS15 treatment in the microfluidic circulatory system.

If SS can elevate the levels of superoxide in both metastatic and non-metastatic cells, then why does it cause more damage to non-metastatic cells? To answer this question, we examined the mitochondria where superoxide was elevated after circulation. It was found that circulation damaged the mitochondria of MCF7 cells by reducing their size and causing the loss of mitochondrial membrane potential (MMP), but these damages were not observed in MDA-MB-231 cells (Figure 4A and 4B, Figure 5A and 5B). These phenomena were also not observed in MCF7 cells placed under non-adhesive and static conditions, indicating that SS is the primary cause of mitochondrial damage in non-metastatic MCF7 cells (Figure 5A and 5B). Furthermore, treating MCF7 cells with the antioxidant PG blocked mitochondrial fragmentation (Figure 5B). Therefore, we propose that SS mainly damages non-metastatic CTCs in the following order and time line: shear stress → ROS generation (≤ 1 hour) → mitochondrial fragmentation (1-3 hours) → loss of MMP (6 hours) → cytochrome c release → caspase activation/apoptosis (8-20 hours) (Figure 5C).

Next, we investigated why SS-produced oxidative stress damaged mitochondria in MCF7 cells but not in MDA-MB-231 cells. For the MCF7 cells, which are sensitive to SS-induced damages, we pre-treated them either with manganese (III) tetrakis (4-benzoic acid) porphyrin chloride (MnTBAP) to simulate the function of MnSOD, which can convert mitochondrial superoxide into hydrogen peroxide [23], or with the antioxidant PG before circulation. Both treatments significantly reduced mitochondrial fragmentation, prevented the loss of MMP, decreased SS-induced apoptosis, and increased cell viability in MCF7 cells (Figure 4A-4D and Figure 5B). Furthermore, pre-treating MDA-MB-231 cells with a superoxide dismutase (SOD) inhibitor, 2-methoxyestradiol (2-ME) [24], caused them to lose MMP after 6 hours of SS treatment (Figure 4A and 4B). These results suggest that antioxidant activity, especially superoxide scavenging, is required for CTCs to counteract SS-mediated mitochondrial damage and cell death.

### High levels of MnSOD protect cancer cells from SS-induced apoptosis

To determine which SOD serves to protect cancer cells against SS-induced cell death, the levels of two superoxide dismutase enzymes were determined, including MnSOD, which is localized in mitochondria (Figure 5D), and CuZnSOD, which is localized in the cytosol. Metastatic 231-C3 cells exhibited 2.6-fold more MnSOD than non-metastatic MCF7-C3 cells, while their levels of CuZnSOD were similar. In addition, the levels of these two enzymes showed little variation during 12 hours of circulation (Figure 4E). To study the role of MnSOD, its expression was repressed using two MnSOD-specific siRNA (siMnSOD-1 and siMnSOD-2) in 231-C3 cells (Figure 4F); subsequently, the cells were subjected to SS15 treatment for 18 hours. Silencing MnSOD expression significantly increased apoptosis and reduced the viability of 231-C3 cells to levels similar with those achieved by using the MnSOD inhibitor 2-ME (Figure 4G and 4H). Furthermore, overexpression of MnSOD in MCF7-C3 cells resulted in a significant reduction of apoptosis and elevation of cell viability during circulation (Figure 4I-4K).

We further substantiated the anti-SS function of MnSOD by selecting the small population of MnSOD-positive MCF7-C3 cells that maintained normal mitochondrial morphology and retained normal MMP after 6 hours of circulation (Figure 5E), and further survived after 18 hours of circulation. These MCF7-S1 cells displayed higher levels of MnSOD, lower rates of apoptosis and higher percentages of cell survival against SS treatment compared with those of their parental MCF7-C3 cells (Figure 5F-5H). All these results support the notions that MnSOD can protect CTCs from SS-induced mitochondrial damage and apoptosis and that the expression of MnSOD is heterogeneous among the populations of MCF7 and MDA-MB-231 cells.

### Heterogeneous expression of MnSOD determines the survivability of CTCs

**Figure 6:**
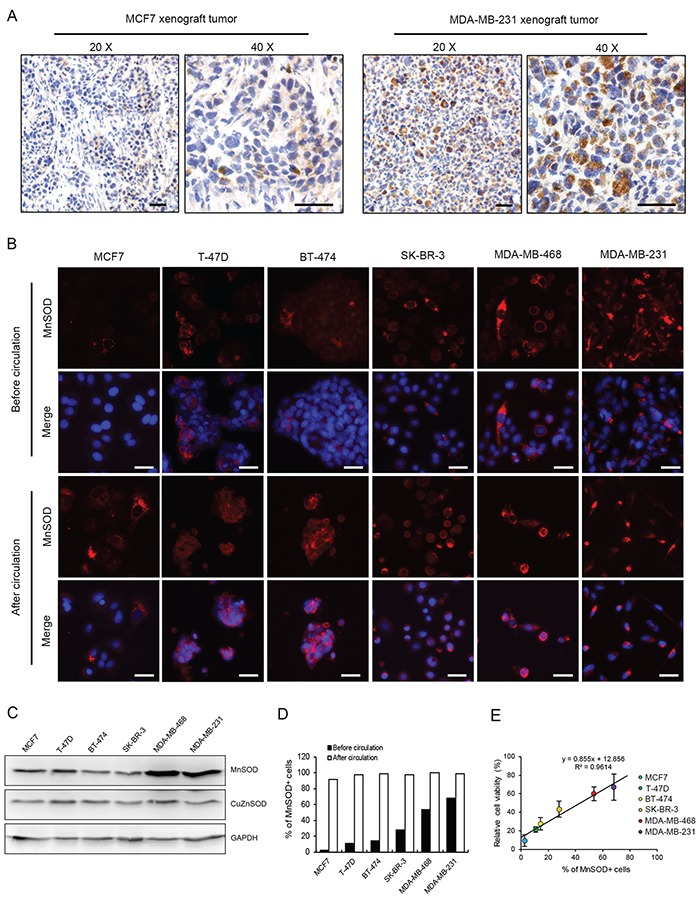
Heterogeneous expression of MnSOD determines the survivability of CTCs **A.** Expression of MnSOD in MCF7 and MDA-MB-231 cells within xenograft tumors was compared by IHC staining. Scale bars represent 50 μm. **B.** Expression levels of MnSOD in six breast cancer cell lines before and after 18 hours of SS treatment were compared by IF staining. Nuclei were stained with Hoechst 33342 (blue). Scale bars represent 20 μm. **C.** Expression levels of MnSOD and CuZnSOD in six breast cancer cell lines were determined by Western blotting. GAPDH was used as a loading control. **D.** The number of MnSOD-positive cells from all six cell lines was determined by analyzing IF staining results. **E.** The survival rates of six breast cancer cell lines determined by the MTT assay after 18 hour circulation were plotted against the percentages of MnSOD+ cells before circulation. The results represent the mean ± SD from three independent experiments.

The heterogeneous expression of MnSOD was further revealed by immunohistochemistry (IHC) staining in xenograft breast tumors derived from MCF7 or MDA-MB-231 cells (Figure 6A). In addition, this heterogeneous expression pattern of MnSOD was also confirmed by immunostaining in all four types of breast cancer cell lines tested in this study, including ER+/HER2-, MCF7 and T-47D; ER+/HER2+, BT474; ER-/HER2+, SK-BR-3; and triple negative, MDA-MB-468 and MDA-MB-231 [25] (Figure 6B). Very importantly, the two highly metastatic cell lines, MDA-MB-468 and MDA-MB-231, both had the highest percentages of MnSOD-positive cells and the highest levels of MnSOD (Figure 6B and 6C).

To determine the correlation between MnSOD expression and cell survival in circulation, all six cell lines were circulated in the microfluidic system under SS15 for 18 hours. Immunofluorescence staining showed that although the percentages of MnSOD-positive cells varied significantly among these cell lines (3-65%), over 90% of the surviving cells were MnSOD positive (Figure 6B and 6D). Significantly, a highly positive linear correlation was found between the number of MnSOD-positive cells existed prior to circulation and the number of viable cells after SS15 treatment (R^2^ = 0.9614) (Figure 6E). Together, these results establish the important role of MnSOD in protecting CTCs from SS-induced apoptosis in circulation.

### High levels of MnSOD increase metastatic potential of breast cancer cells

**Figure 7:**
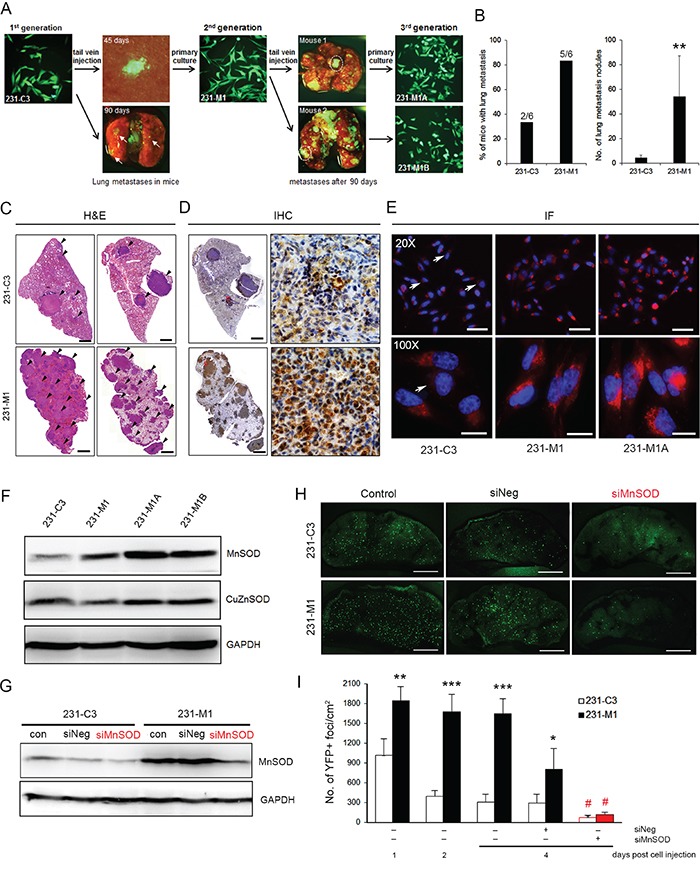
High levels of MnSOD increase metastatic potential of breast cancer cells **A.** Flowchart illustrating the steps of generating 231-C3 series cells with increased metastatic ability to form lung metastases in nude mice. **B** and **C.** Metastatic ability of the 231-C3 series cells was assessed by counting the number of lung metastases after cell injection. The percentage of mice with lung metastases was calculated from all lung leaves (B, left). The number of lung metastatic nodules was calculated from the left lobe of the lung in all six mice (B, right). Representative pictures of H&E-stained lung metastases (indicated by black arrows) 90 days after the injection of 231-C3 or 231-M1 cells (C). ^**^P < 0.01 by Student's t test. Scale bars represent 1 mm. **D.** IHC analysis of MnSOD expression in lung metastases 90 days after the injection of 231-C3 or 231-M1 cells at lower (left) and higher magnifications (right). Scale bars represent 1 mm. **E.** IF staining of MnSOD expression in the 231-C3 series cells observed under 20X and 100X objectives. Cells with lower levels of MnSOD are indicated by white arrows. Scale bars represent 10 μm. **F.** Expression of MnSOD and CuZnSOD in the 231-C3 series cells was determined by Western blot analysis. GAPDH was used as a loading control. **G.** Knockdown efficiency of MnSOD in 231-C3 and 231-M1 cells was examined by Western blotting. **H** and **I.** Effects of siRNA-#1-mediated MnSOD knockdown on metastasis were determined using a mouse lung metastasis model. Representative YFP images (H) and quantification of YFP-positive foci (I) of 231-C3 and 231-M1 cells on the lung surface 1-4 days post injection with or without siRNA transfection. Scale bars represent 2 mm. ^*^P < 0.05, ^**^P < 0.01, ^***^P < 0.001 using Student's *t* test: 231-M1 *vs*. 231-C3.^#^*P* < 0.05 comparing with siNeg groups.

Next, we examined whether high levels of MnSOD can increase the metastatic capacity of breast cancer cells by establishing three generations of 231-C3 cells (Figure 7A). 231-M1 cells were isolated from lung micrometastases on the 45 days after 231-C3 cells were injected into the tail vein of nude mice (Figure 1E). These 231-M1 cells were considered to be more malignant than the 231-C3 cells, as many more lung metastases with much larger tumor sizes were formed in five out of six mice after the mice received an injection of 231-M1 cells into their circulatory system. Two cell lines were further isolated from two 231-M1 metastatic tumors, namely, 231-M1A and 231-M1B (Figure 7A-7C). IHC analysis showed that 231-M1 lung metastatic tumors had much higher and more uniform expression of MnSOD than 231-C3 lung metastasis (Figure 7D). The 231-M1A and M1B cells displayed even more homogenous and higher expression of MnSOD than did the 231-M1 and 231-C3 cells (Figure 7E and 7F).

**Figure 8:**
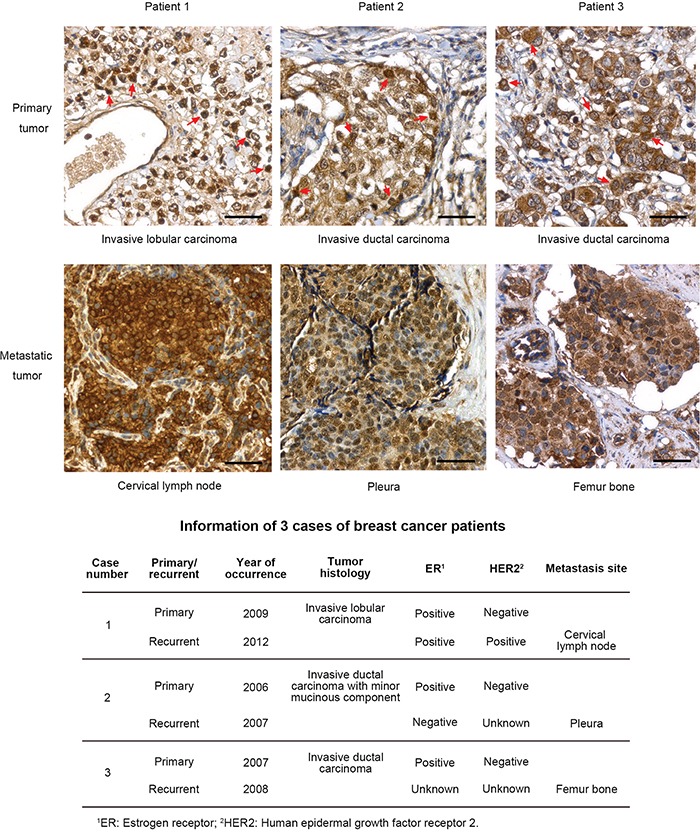
Recurrent human breast tumors have more homogenous expression of MnSOD Formalin-fixed, paraffin-embedded samples of primary tumors and distant recurrent metastases from three patients with invasive breast carcinoma were analyzed for MnSOD expression by IHC staining. Cells with high expression of MnSOD in the primary tumors were indicated with red arrows. Scale bars represent 100 μm. Patient information of the three cases of breast cancer with primary and recurrent tumors were list out in the lower panel.

We also compared the levels of MnSOD between primary and metastatic human breast tumors. Three cases of invasive breast cancer were identified, and samples of both primary and metastatic tumors that had formed at the cervical lymph node, pleura and femur bone after surgical excision of the primary tumors were obtained. IHC analysis of formalin-fixed, paraffin-embedded tissue samples revealed that the metastatic tumors displayed a higher and more homogenous expression of MnSOD than did the primary tumors, in which only some of the tumor cells displayed high MnSOD staining (Figure 8, red arrows). The patients' information is summarized in Figure 8.

To validate the role of MnSOD in supporting the survival and metastasis of CTCs, the expression of MnSOD was silenced using siRNA-#1 in 231-C3 and 231-M1 cells before injecting them into the tail vein of nude mice. SiRNA-mediated knockdown of MnSOD significantly attenuated the metastatic ability of 231-M1 cells to the same low level exhibited by 231-C3 cells (Figure 7H and 7I). These results suggest that MnSOD plays a very important role during metastasis by increasing the survival of breast cancer cells in the circulation.

### High levels of MnSOD confer resistance to DOX and knockdown of its expression sensitizes cells to DOX treatment

**Figure 9:**
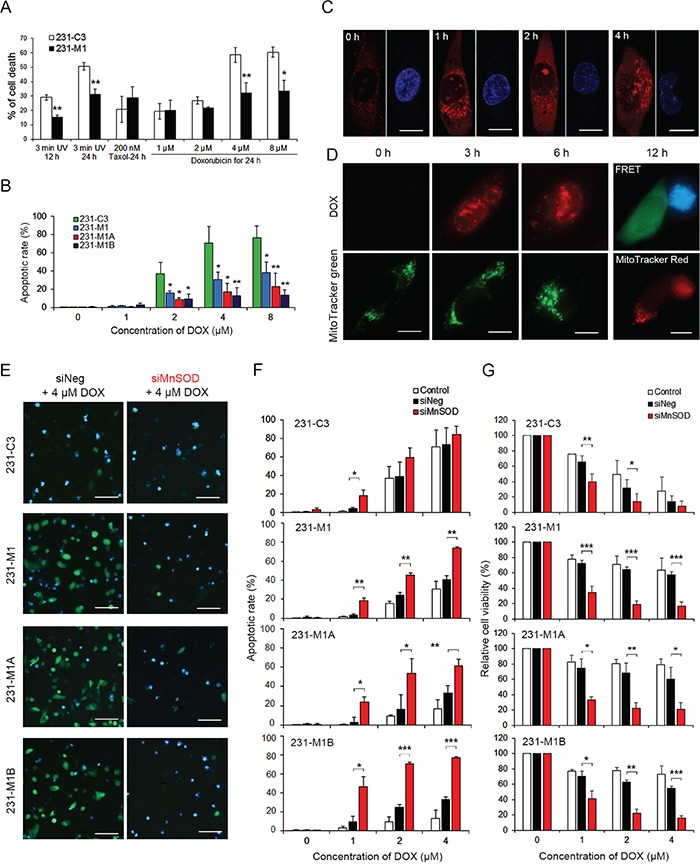
High levels of MnSOD confer resistance to DOX and knockdown of its expression sensitizes cells to DOX treatment **A.** Effects of three death inducers, 3 minutes of UV irradiation, Taxol and DOX, on 231-C3 and 231-M1 cells were determined using the MTT assay. ^*^P < 0.05, ^**^P < 0.01 using Student's *t* test: 231-M1 *vs*. 231-C3 cells. **B.** Apoptotic effects of DOX on 231-C3 series cells were quantified by FRET imaging analysis. **C.** DOX-mediated production of mitochondrial superoxide was determined by staining MDA-MB-231 cells with 5 μM MitoSOX. Nuclei were stained with Hoechst 33342 (blue). Scale bars represent 5 μm. **D.** Effects of DOX (4 μM, red) on mitochondrial morphology in MDA-MB-231 cells were revealed by staining cells with 100 nM MitoTracker Green (green). Live cells (green) and apoptotic cells (blue) were revealed by FRET imaging and their mitochondrial morphologies were revealed by staining cells with 100 nM MitoTracker Red. Scale bars represent 5 μm. **E-G.** 231-C3 series cells were transfected with negative siRNA (siNeg) or MnSOD siRNA-#1 (siMnSOD) and treated with 0, 1, 2, 4 μM DOX for 24 hours. FRET images of sensor cells treated with 4 μM DOX (E). Scale bars represent 100 μm. Apoptotic rates of these sensor cells were quantified by FRET imaging (F), and cell viability was measured using the MTT assay (G). The results represent the mean ± SD from three independent experiments. ^*^P < 0.05, ^**^P < 0.01, ^***^P < 0.001 using Student's *t* test comparing with the control group or as otherwise indicated in the graphs.

Since MnSOD can scavenge superoxide, we postulated that the 231-C3 series cells, with high levels of MnSOD, may have a stronger resistance to ROS-generating agents, such as DOX and radiation. The 231-C3 series cells were treated with 3 minutes of UV irradiation, 1-8 μM DOX, or 200 nM Taxol. The results showed that 231-M1 cells exhibited higher resistance to DOX and UV irradiation-induced cell death than did 231-C3 cells, but no higher resistance was observed when these cells were treated with Taxol, which does not generate ROS (Figure 9A). Furthermore, 231-M1A and 231-M1B cells, with even higher levels of MnSOD, displayed lower rates of apoptosis compared with 231-M1 and 231-C3 cells during DOX treatment (Figure 9B). In particular, after being treated with 8 μM DOX for 24 hours, the apoptotic rates among these four cell lines were 76.7% (231-C3), 38.3% (231-M1), 22.8% (231-M1A), and 13.3% (231-M1B) (Figure 9B). We confirmed that 4 μM DOX produced superoxide after 1 hour of drug treatment (Figure 9C), damaged mitochondria after 3-6 hours of drug treatment (Figure 9D), and caused MMP reduction and activated caspase-3-dependent apoptosis after 12 hours (Figure 9D).

SiRNA-mediated MnSOD knockdown significantly increased the DOX-induced apoptosis in M1, M1A and M1B cells expressing high levels of MnSOD. This was especially significant for M1A and M1B cells treated with 2-4 μM DOX for 24 hours, in which the apoptotic rates were 2-3-fold higher in the MnSOD siRNA-#1 group compared with those of the control siRNA group (Figure 9E and 9F, and Supplementary Figure S4). Even at a low DOX concentration of 2 μM, silencing MnSOD successfully increased the efficacy of DOX-induced apoptosis to more than 50% (Figure 9F) and reduced cell viability to less than 20% in all four cell lines (Figure 9G). These results suggest that metastatic breast cancer cells can develop DOX resistance by overexpressing MnSOD and this resistance can be overcome by reducing MnSOD expression.

## DISCUSSION

**Figure 10:**
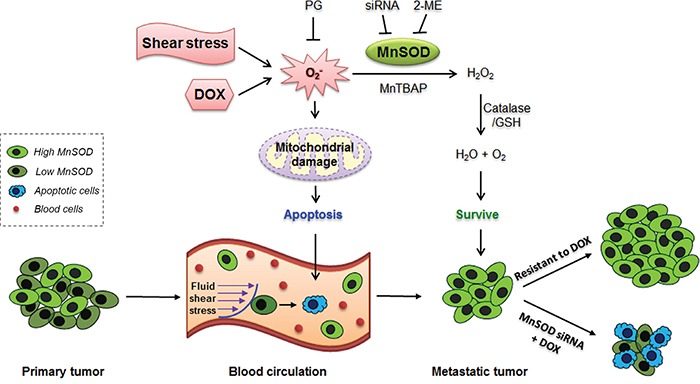
Proposed mechanism of MnSOD in supporting cancer cell survival, metastasis and DOX resistance Both shear stress and DOX can damage CTCs by producing superoxide, which can damage mitochondria and induce apoptosis. Only the CTCs with high levels of MnSOD can resist SS-induced cell death as MnSOD can scavenge superoxide. When the surviving CTCs grow into metastatic tumors, the tumor cells will continue to express high levels of MnSOD, which confer resistant to DOX. Knockdown of MnSOD sensitizes resistant metastatic breast cancer cells to DOX treatment.

In this study, we discovered an interconnection between three major events in cancer development and treatment: CTC survival, metastasis, and resistance to the first-line anti-cancer agent DOX (Figure 10). These three events are unlikely to occur independently, but they may stem from one common origin, which is a high expression level of MnSOD. We propose that when cancer cells enter into the circulation and become CTCs, the fluid SS present in the human artery can increase the levels of superoxide, which damages mitochondria. For CTCs with lower levels of MnSOD, they can be killed by SS-induced apoptosis in circulation. However, cells with higher levels of MnSOD can survive SS-induced apoptosis and further develop into metastatic tumors. This explains why MnSOD expression is both more uniform and higher in metastatic tumors formed in mice (Figure 7D), in malignant tumors of breast cancer patients (Figure 8), and in the results of previous clinical studies [26–28]. We further confirmed that this high expression of MnSOD can be inherited by CTC progeny during metastasis. For example, the 231-C3 series cells isolated from metastatic tumors exhibited higher levels of MnSOD compared with their parental cells (Figure 7A-7F).

MnSOD is the most important antioxidant enzyme, and in some earlier studies, it was found to suppress tumor formation [29–31]. However, recent studies have suggested that MnSOD could enhance the malignancy of tumor cells by promoting their resistance to anoikis [32] or by sustaining the Warburg effect [33]. In this study, we found a new integrative role of MnSOD in supporting breast cancer CTC survival, metastasis and resistance to DOX. We made these discoveries by applying integrative model systems that overcame the technical limitations in studying cancer cells in the circulation and detecting apoptosis in real time. For example, a novel microfluidic circulatory system was developed to produce physiologically relevant shear forces, which is better than the previously reported SS-generating methods using corn-plate viscometers [5, 34, 35] or syringe needles [4]. Both the zebrafish tumor model [36, 37] and the experimental metastatic mouse model [38] were optimized in this study. We then introduced a real-time apoptosis sensor into these systems. By doing so, we found for the first time that cancer cells circulating in zebrafish, or in a microfluidic circulatory system, could be killed by SS-induced apoptosis (Figure 2 and Figure 3). We also observed that many cancer cells died via apoptosis in the lung 1-2 days after having been injected into the circulation of nude mice (Figure 1). More importantly, we found that metastatic breast cancer cells displayed a stronger ability to survive SS-induced apoptosis because they expressed higher levels of MnSOD (Figure 4-Figure 7).

MnSOD is specifically localized in mitochondria, where superoxide molecules are constantly generated as by-products during the production of ATP [39]. Under normal conditions, superoxide is retained within the inner membrane of mitochondria, where MnSOD can convert it to the less toxic hydrogen peroxide, which can then diffuse into the cytosol and be converted by catalase into oxygen and water. Mitochondria usually maintain a filamentous morphology in attached cells as they can bind to microtubules. When cells detach and circulate, the filamentous structure of microtubules will be affected [40], which may trigger mitochondria to produce more superoxide. In this study, we found that superoxide levels quickly increased in mitochondria after cells had been circulating for less than 60 minutes. This was followed by a chain of events, including mitochondrial fragmentation (1-3 hours), loss of MMP (6 hours) and caspase-dependent apoptosis (8-20 hours), only in non-metastatic MCF7 cells (Figure 5C) but not in metastatic 231-series cells. High levels of MnSOD in the mitochondria were able to scavenge the SS-elevated superoxide, thus allowing the metastatic cells to survive the oxidative stress-induced apoptosis during circulation.

Another important finding of this study is that high levels of MnSOD confer resistance to the anti-cancer agent DOX, which can produce superoxide (Figure 9) [41–43]. Reducing MnSOD expression using specific siRNA or inhibiting its activity by using 2-ME increased the destructive effect of fluid SS (Figure 4). Most importantly, siRNA-mediated knockdown of MnSOD significantly increased the efficacy of DOX (Figure 9). These results identify MnSOD as a novel marker for detecting metastatic CTCs and a potential therapeutic target for killing metastatic breast cancer cells.

## MATERIALS AND METHODS

### Cell culture

Breast cancer cell lines MCF7, BT-474, SK-BR-3 and MDA-MB-468 were purchased from the ATCC. T-47D and MDA-MB-231 were provided by Prof. Xiaofeng Le from University of Texas, M.D. Anderson Cancer Center (Houston, USA). Cell Line Authentication Services were performed using short tandem repeat DNA profiling analysis (Genetica DNA Laboratories, USA). MCF7 cells were cultured in MEM, BT-474 cells were cultured in RPMI 1640, and the other four cell lines were cultured in DMEM (Invitrogen, USA) with 10% fetal bovine serum (HyClone, UK) and 1% penicillin-streptomycin (Gibco, USA). MCF7 and MDA-MB-231 cells were transfected with the sensor C3 plasmid using Lipofectamine 2000 (Invitrogen, USA). After selection with 500 μg/mL G418 (PAA, Germany), single-cell colonies expressing the C3 sensor were designated MCF7-C3 and 231-C3 cells (Figure 1B) [17].

### Experimental lung metastasis model and orthotopic xenograft model in nude mice

All mouse experiments were approved by the Institutional Animal Care and Use Committee (IACUC) of Nanyang Technological University (NTU). In the experimental lung metastasis model, a total of 5 × 10^5^ MCF7-C3 or 231-C3 cells were injected into the tail vein of 6-8-week-old female BALB/c nude mice (BioLasco Company, Taiwan). After the mice were sacrificed at the designed times, various tissues were examined for micrometastases using an MVX10 Fluorescence MacroZoom System (Olympus, Japan) equipped with FRET filters (Ex = 436 ± 10 nm; diachronic mirror = 455 nm; Em_1_ = 480 ± 20 nm and Em_2_ = 535 ± 15 nm).

Orthotopic xenograft models were generated by injecting of 1 × 10^6^ MCF7 or MDA-MB-231 cells in 100 μL of Matrigel (BD Biosciences, USA) into the mammary fat pad of nude mice. For mice injected with MCF7 cells, one estrogen pellet (1.7 mg per pellet, Innovative Research of America, USA) was subcutaneously implanted into each mouse 4 days prior to cell injection. Metastatic 231-C3 series cell lines were isolated from micrometastases or macrometastases on lung by dissection under imaging system followed by collagenase IV digestion (Sigma) (Figure 7A).

### Microinjection and imaging of sensor cells in zebrafish circulation

All zebrafish experiments were approved by the IACUC of NTU. 72 hours post fertilization larvae were anesthetized with 0.01% tricaine and positioned on a wet agarose microinjection pad. 100-200 cancer cells were injected into zebrafish larva at 72 hours post fertilization. Zebrafish imaging and extravasation analyzing were performed as described previously [37]. Images of cancer cells and zebrafish were captured using a motorized fluorescence microscope (Axio Observer Z1, Carl Zeiss, Germany) equipped with the aforementioned FRET filters, a computer-controlled camera (AxioCam MRm, Carl Zeiss, Germany) and operated by Zen 2012 software (Carl Zeiss). The digital fluorescence images were then processed using Image-Pro Plus software (Media Cybernetics, USA) to calculate the FRET effects for determining the apoptotic state of sensor cells (Supplementary Figure S1). Zebrafish heartbeat was counted in a 35.5°C chamber under a microscope (Axio Observer Z1). The same group of fish (*n* = 5) was analyzed at different time points, from 4 to 24, as well as 48 and 72 hours post injection, and the average heart rates were calculated.

### Detection of apoptosis in a microfluidic circulatory system

A microfluidic circulatory system (Figure 3A) was assembled by using a peristaltic pump (Ismatec, Germany) to generate a pulsatile flow in a circulatory silicone tubing (Ismatec, Germany) with a diameter of 500 μm and a total length of 1.5 m. A polydimethylsiloxane (PDMS)-based observation chip was linked with the microfluidic circulatory system when live cell imaging microscopy was conducted. SS in the tubing was calculated using Poiseuille's equation [4], τ = 4Qη/πR^3^, where τ is SS in dyne/cm^2^, Q is flow rate in cm^3^/s, η is the dynamic viscosity of the fluid (the culture medium can be treated as water at 37°C; η = 0.01 dyne*s/cm^2^), and R is the radius of the silicone tubing (250 μm). The flow rate can be adjusted to achieve SS range from 5 to 30 dyne/cm^2^. To prevent cells attaching to the tubing or the PDMS chip, the whole system was pre-coated with 0.5% Pluronic F127 in PBS (Invitrogen, USA) for 1 hour at room temperature before the experiments. A Pluronic F127-coated, 96-well plate was also used as a negative control for the no SS condition.

Cancer cells were collected using 0.05% trypsin containing 0.53 mM EDTA (Gibco, USA), washed twice with PBS and re-suspended in fresh culture medium at a cell density of 2 × 10^5^/ml before injected into the microfluidic system. One milliliter of cell suspension was added to the microfluidic circulatory system and subjected to circulation for varying times at 37°C in a humidified CO_2_ incubator. For live cell imaging, the pump was switched off to stop the medium flow, and the movement of sensor cells was retained within the observation chip by closing the two control valves. The rate of apoptosis was determined by FRET imaging microscopy.

### Determination of ROS levels, mitochondrial morphology and membrane potential of cells

ROS levels were determined by staining cells with 10 μM CM-H_2_DCFDA (Invitrogen, USA), and mitochondrial superoxide levels were determined by staining cells with 10 μM MitoSOX Red (Invitrogen, USA) following manufacturer's instruction. Mitochondrial morphology and membrane potential of cells were determined by staining cells with 100 nM MitoTracker Red-CMXRos (Life Technologies, USA) following manufacturer's instruction. Fluorescence-emitting dichlorofluorescein (DCF) were recorded using a fluorescence microscope (Axio Observer Z1). Mitochondria stained with MitoTracker Red-CMXRos were imaged with a LSM710 confocal microscope (Carl Zeiss, Germany). MitoSOX fluorescence was measured immediately using a flow cytometer (LSRII, BD Biosciences, USA).

### Western blot analysis

Cells were collected with or without treatments, lysed in RIPA buffer, electrophoreses on SDS-PAGE and transferred to PVDF membrane. Protein blots were probed with primary antibodies against MnSOD at 1:3,000 (Abcam, UK), CuZnSOD at 1:1,000 (Cell Signaling, USA), and GAPDH at 1:1,000 (Cell Signaling, USA), followed by secondary goat anti-rabbit IgG incubation at 1:5,000 (Bio-Rad) for 1 hour at room temperature. The blots were developed using ECL solutions (Thermo Scientific, USA).

### Immunofluorescence, immunohistochemistry and H&E staining

For immunofluorescence staining, cells were cultured in a 12-well removable chamber (Ibidi, Germany). The cells were fixed with 4% paraformaldehyde for 15 minutes, permeabilized with 0.2% Triton X-100 for 20 minutes, blocked with 3% BSA in PBS containing 0.3 M glycine for 30 minutes and incubated with rabbit anti-MnSOD antibody (Abcam, UK) at a 1:100 dilutions in PBST containing 3% BSA overnight at 4°C. The cells were further incubated with TRITC-conjugated secondary antibody (Calbiochem, USA) at a 1:100 dilutions for 1 hour at room temperature. Nuclei were stained with Hoechst 33342 before the slides were mounted with Mowiol^®^ 4-88 (Calbiochem, USA).

Immunohistochemistry (IHC) analysis and hematoxylin and eosin (H&E) staining were performed on 5-μm paraffin sections of experimental lung metastases using standard protocols (Leica). For the IHC staining, antigens were retrieved using heated citrate buffer at 100°C for 5 minutes. For the orthotopic xenograft tumors, 5-μm cryosections were directly fixed with 4% paraformaldehyde for 30 minutes. The slides were then blocked and stained using the Rabbit-Specific HRP/DAB Detection IHC Kit (Abcam, UK) according to the manufacturer's instructions. The primary rabbit anti-MnSOD antibody (Abcam) was diluted 1:100 in PBST containing 3% BSA and incubated overnight at 4°C. All color images of the IHC analysis and H&E staining were recorded using a color camera (AxioCam 506, Carl Zeiss).

### Downregulation and inhibition of MnSOD

Validated Silencer Select siRNA against MnSOD (siRNA No.1: ID s13268; siRNA No.2: ID s13269) and Silencer Select Negative Control No. 1 siRNA were purchased from Ambion (Life Technologies, USA). Before the cells were transfected, 1 × 10^6^ cells were seeded into 60-mm dishes. After 24 hours, the cells were washed, and the media was replaced with OptiMEM (Life Technologies). The cells were then transfected with 20 nM siRNA using Lipofectamine RNAiMAX (Life Technologies), according to the manufacturer's instructions. After the overnight incubation, the transfection complex was replaced with regular complete DMEM without antibiotics. After 48 hours, the cells were transfected with siRNA again using the same procedure. After an additional 48 hours (i.e., 96 hours after the first transfection), the cells were harvested for further experiments. To inhibit the activity of MnSOD, 20 μM 2-methoxyestradiol (Sigma-Aldrich) was used to pre-treat the cells for 1 hour before they were introduced into the microfluidic circulatory system.

### Overexpression of MnSOD in MCF7 cells

The pMnSOD plasmid was purchased from Origene (USA). This plasmid was constructed with a pCMV6-Entry vector, and MnSOD was tagged with Myc-DDK at the C-terminal. The MCF7-C3 cells were transiently transfected with pMnSOD plasmid or an empty vector using lipofectamine 3000 (Life Technologies, USA), according to the manufacture's protocol. Briefly, 1 × 10^6^ cells were seeded into 60-mm dishes. After 24 h, the cells were washed and the media was replaced with serum-free media. Cells were then transfected with 2 μg MnSOD or vector plasmid DNA plus 5 μl lipofectamine 3000 transfection reagent (Life Technologies). 6 h later, the transfection medium was replaced with fresh medium containing 10% fetal bovine serum and 1% penicillin-streptomycin. After transfection for 48 h, the cells were harvested for further experiments.

### Measurement of cell viability by MTT assay

100 μl of cell suspension was added to each well of a 96-well plate followed by the addition of 10 μl 3-(4,5-dimethylthiazol-2-yl)-2,5-diphenyltetrazolium bromide (MTT, Sigma-Aldrich) solution (5 mg/mL). Three hours later, the formazan was solubilized by the addition of 100 μl of a 10% SDS solution containing 0.01 M HCl and an overnight incubation. The optical density at 595 nm was determined using a plate reader (Perkin-Elmer, USA).

### Statistical analysis

All data are represented as the mean ± SD from three independent experiments. Statistical significance was analysed using one-tailed Student's *t* tests, and ^*^P < 0.05 was considered significant.

## SUPPLEMENTARY MATERIALS FIGURES AND VIDEOS








