# Supplementary material for: High expression of MnSOD promotes survival of circulating breast cancer cells and increases their resistance to doxorubicin
Source: Oncotarget. 2016 Jul 1;7(31):50239–57. doi: 10.18632/oncotarget.10360 (PMC5226580; doi:10.18632/oncotarget.10360)
Supplement: Supplementary file 1 [file oncotarget-07-50239-s001.pdf]

High expression of MnSOD promotes survival of circulating breast cancer cells and increases their resistance to doxorubicin

SUPPLEMENTARY FIGURES AND VIDEOS

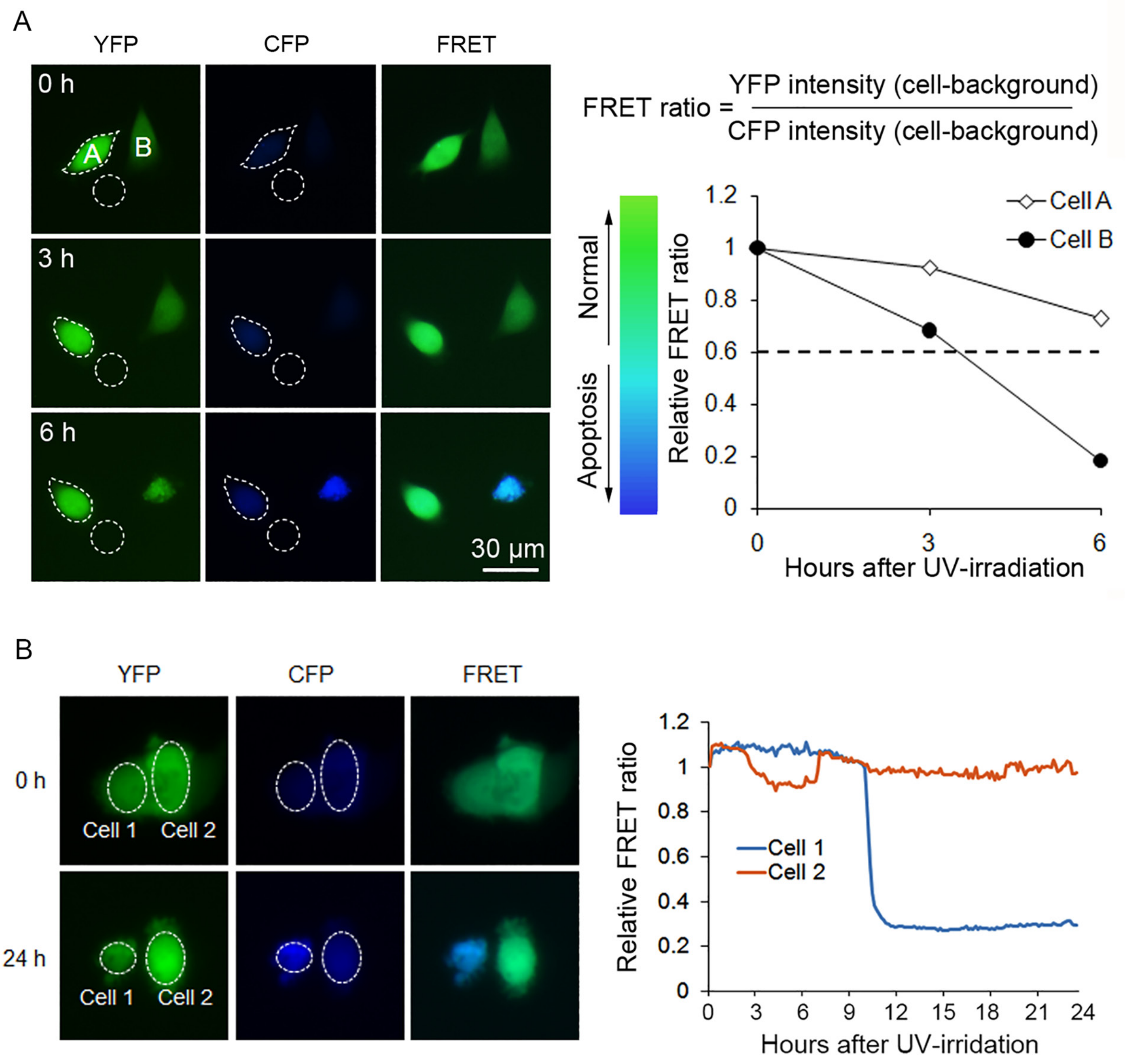

Supplementary Figure S1: Using FRET imaging to quantify apoptosis in sensor cells. A. Apoptosis was induced in 231-C3 cells by 3 minutes of UV irradiation. Fluorescent images of 231-C3 cells were separately obtained from YFP (Em = 535 ± 15 nm) and CFP (Em = 480 ± 20 nm) channels excited under the wavelength for CFP (Ex = 436 ± 10 nm), and the two images were merged to produce a FRET image. The green FRET images indicate live cells, and blue FRET images indicate apoptotic cells. The FRET ratio was calculated from the fluorescence intensity of YFP and CFP from the circled areas. To compare the apoptotic rates of different cells at various times, the relative emission ratio of YFP/CFP in each cell was calculated by normalizing the initial FRET ratio to 1.0 at T_0. Cells were considered to be undergoing apoptosis if their relative FRET ratio was reduced from 1.0 to less than 0.6, which is equivalent to more than a 50% reduction of the FRET effect. **B.** Real-time FRET imaging was conducted to monitor the apoptosis of MCF7-C3 cells after 3 minutes of UV irradiation. Decreases in the YFP/CFP ratio were detected using the same method as in (A).

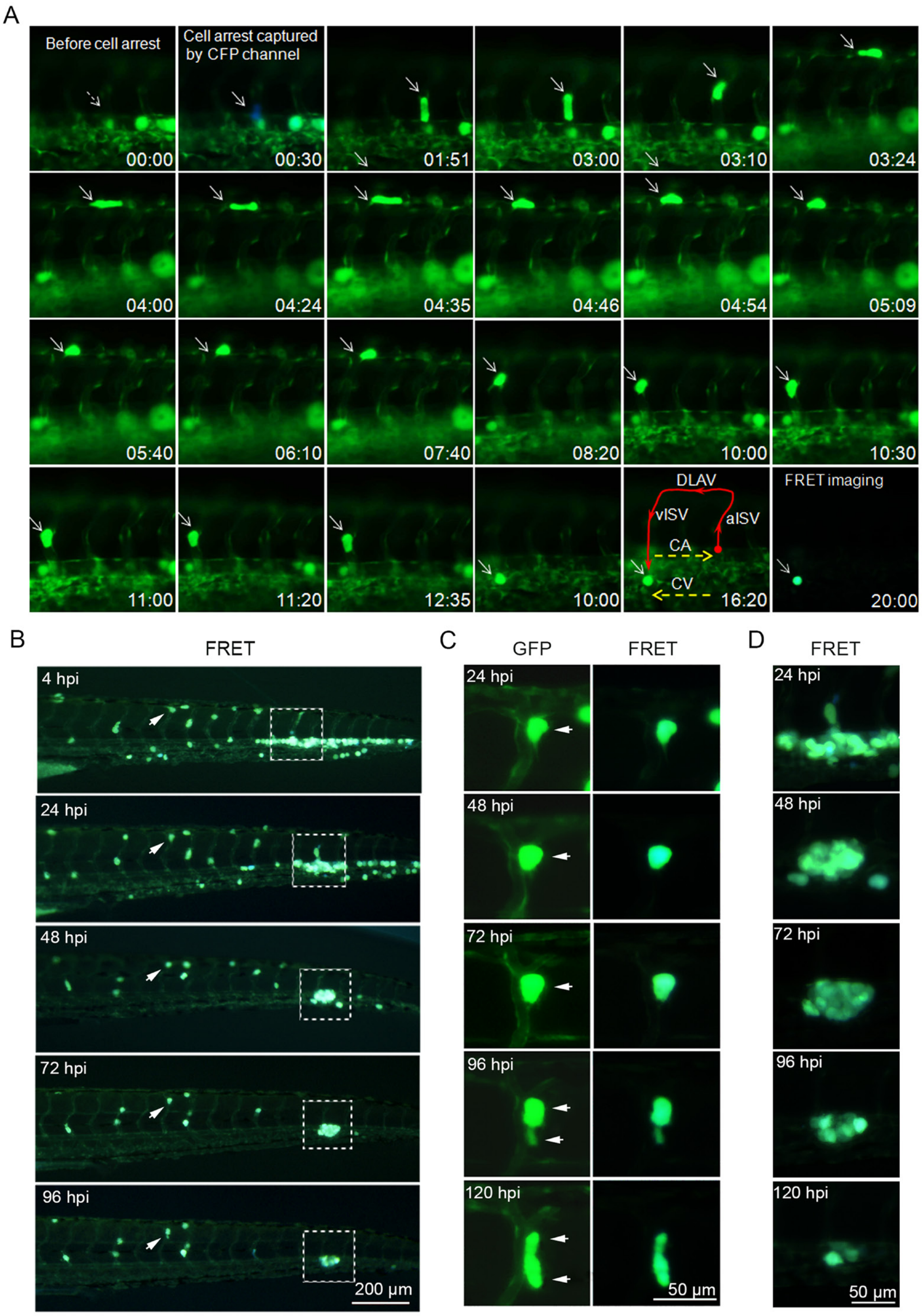

Supplementary Figure S2: Metastatic cancer cells resist hemodynamic SS in zebrafish circulation. A. Travel route of cancer cell in small vessels of a zebrafish. A MCF7-C3 cell was trapped by a small ISV. The cell migrated from the aISV to the DLAV and vISV following the blood flow (direction indicated by yellow arrows), and it finally returned to the circulation in the CV within 16 minutes (travel route indicated by red arrows). The observed cell was still alive after having traveled through the small vessels, as it appeared to be green in the corresponding FRET images. Time is shown as minutes and seconds. **B.** The distribution of 231-C3 cells in zebrafish circulation was continuously observed in the same fish using FRET imaging for 96 hours post injection. **C.** A 231-C3 cell divided in a zebrafish body after extravasation between 96 and 120 hours post injection. **D.** 231-C3 cells resisted SS and survived longer within large vessels by forming an aggregate.

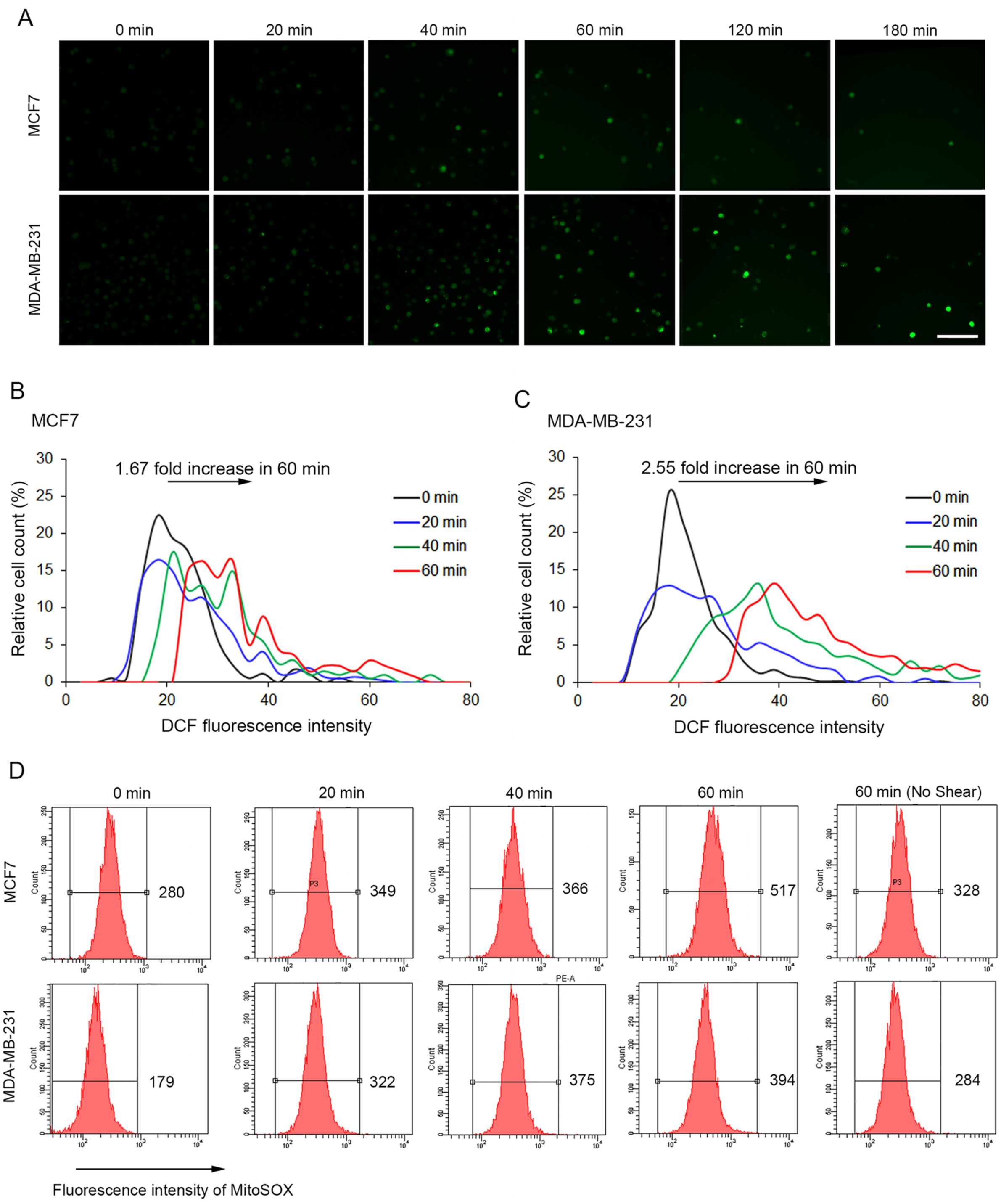

Supplementary Figure S3: Fluid SS promotes ROS generation in circulating cancer cells in less than one hour. A. Production of H_2O_2 in MCF7 and MDA-MB-231 cells was monitored by pre-staining cells with 10 μM CM-H2DCFDA. Cells with positive DCF staining were captured after SS15 treatment for 0, 20, 40, 60, 120 and 180 minutes. Scale bar represents 100 μm. **B** and **C.** The levels of ROS in MCF7 (B) and in MDA-MB-231 (C) cells were determined by measuring the fluorescence intensity of DCF in each cell. The fluorescence intensities of more than 200 cells were used to generate these distribution curves. **D.** Generation of superoxide in mitochondria of MCF7 and MDA-MB-231 cells under SS15 treatment was quantified by MitoSOX; 10,000 cells were analyzed by flow cytometry in each condition, and the calculated average intensity of MitoSOX is shown in each graph.

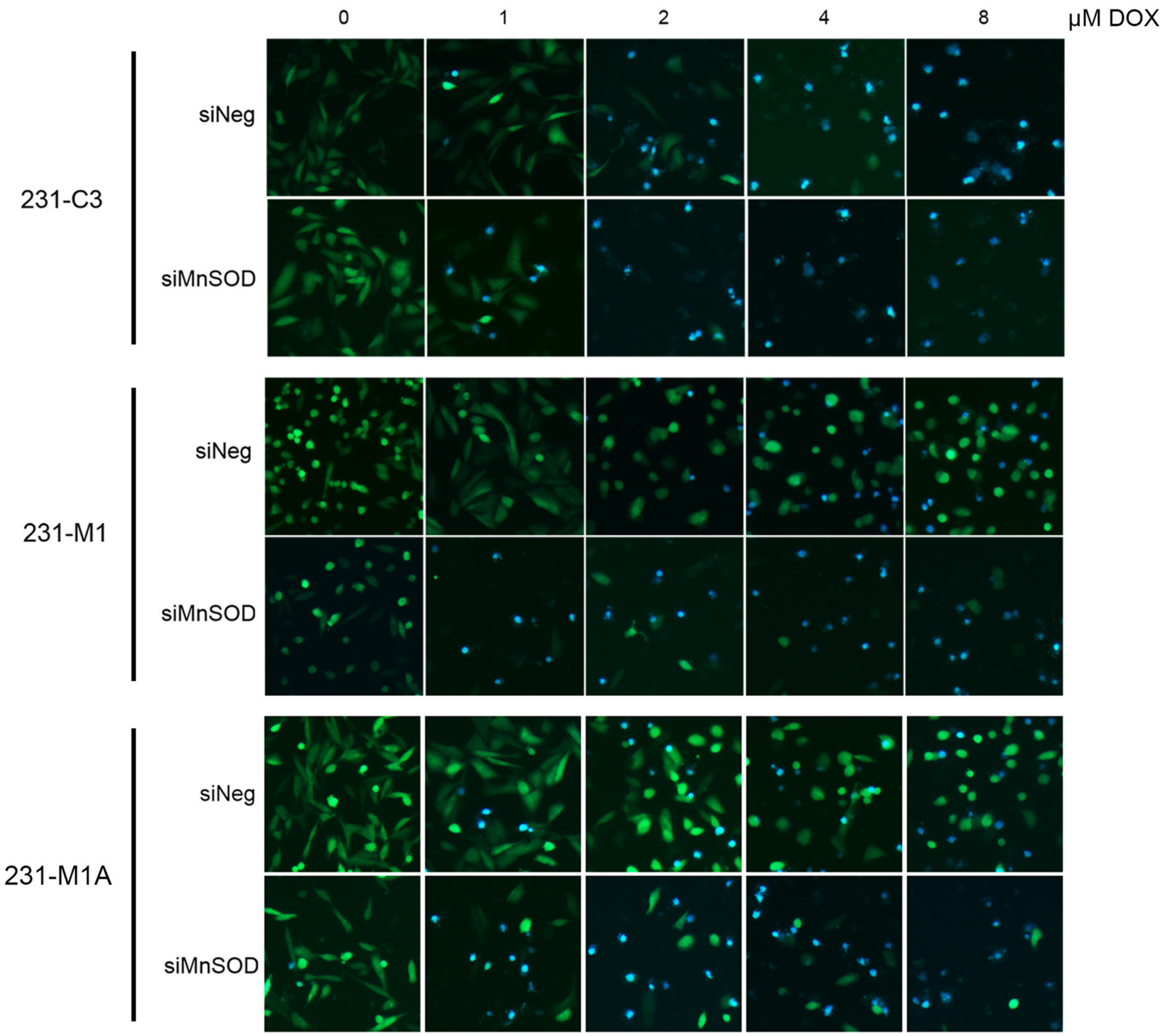

Supplementary Figure S4: Knockdown of MnSOD sensitizes metastatic breast cancer cells to DOX treatment. 231-C3, 231-M1 and 231-M1A cells were treated with 0, 1, 2, 4, 8 μM DOX after being transfected with negative siRNA (siNeg) or MnSOD siRNA-#1 (siMnSOD). Apoptosis was detected using FRET imaging 24 hours after the treatment.

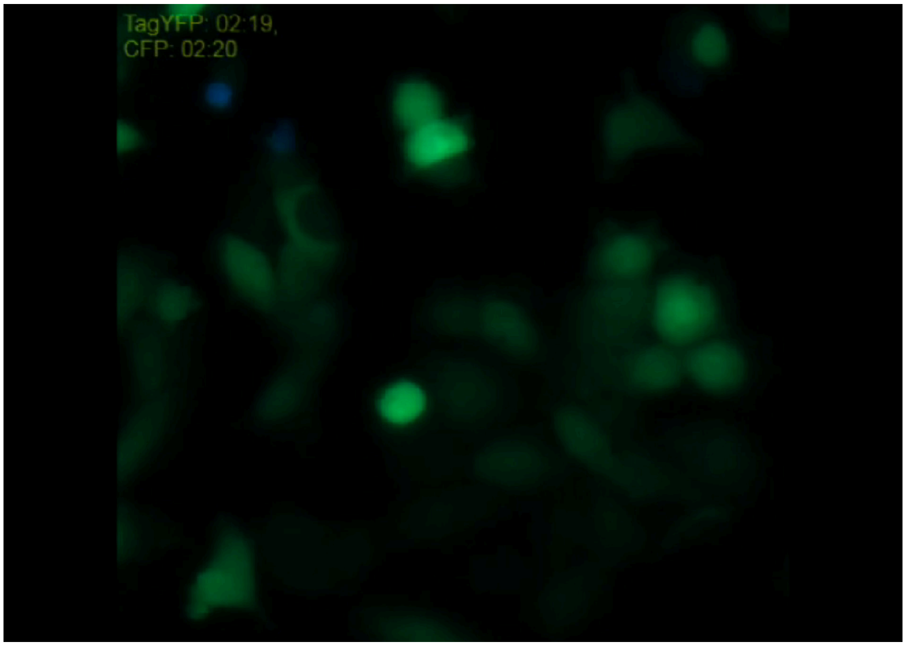

Supplementary Video S1: Real-time detection of apoptosis by FRET imaging of caspase-3/-7 activation. Apoptosis was induced in MCF7-C3cells by 3 minutes of UV irradiation. Time-lapse images were recorded by merging the signals from the CFY and YFP channels. The CFP channel was recorded using an excitation filter of 436 ± 10 nm and an emission filter of 480 ± 20 nm. The YFP channel was recorded using the same excitation filter but an emission filter of 535 ± 15 nm. The living cells appeared green in color, while the apoptotic cells changed color from green to blue in the video. The time line of YFP and CFP channel was shown in the upper left of the video (h:min).

See Supplementary File 1

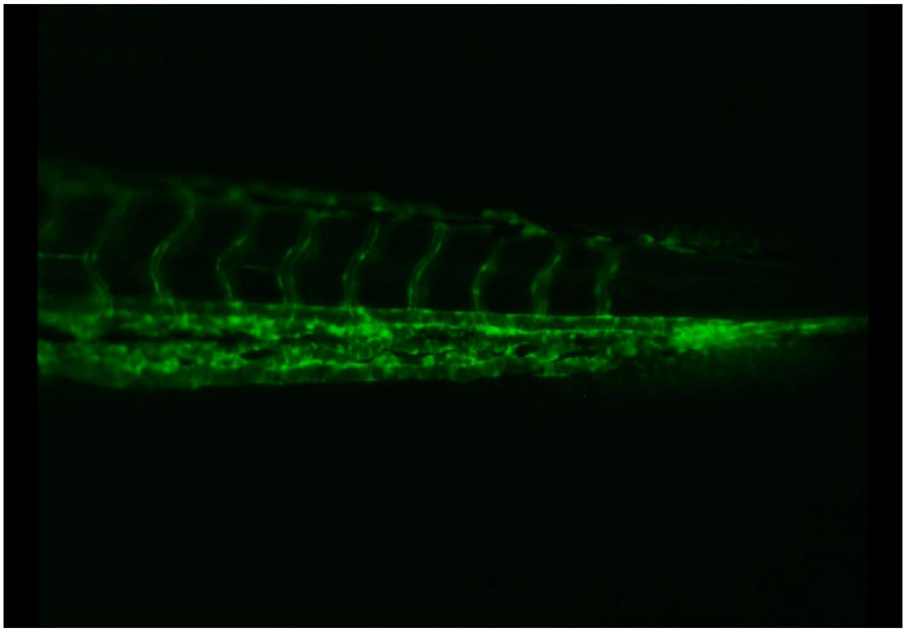

Supplementary Video S2: Blood flow in zebrafish tail region. A 3-dpf zebrafish was mounted in 0.8% agarose and imaged by time-lapse microscopy using an inverted fluorescence microscope (Axio Observer Z1, Carl Zeiss, Germany). The temperature was maintained at 35.5°C using a heating stage. Frames were taken every 20 milliseconds for 12 seconds. The video shows that the blood flowed in a pulsatile manner in the large CA artery located underneath the ISVs, with an estimated heart rate of 175 beat per minute (bpm). In contrast, in large veins, including the CV, and small vessels, such as the aISV, DLAV and vISV, the blood flowed at lower speeds without pulsing. Fast and pulsatile flow in the artery was postulated to generate severe hemodynamic SS on the injected sensor cells.

See Supplementary File 2

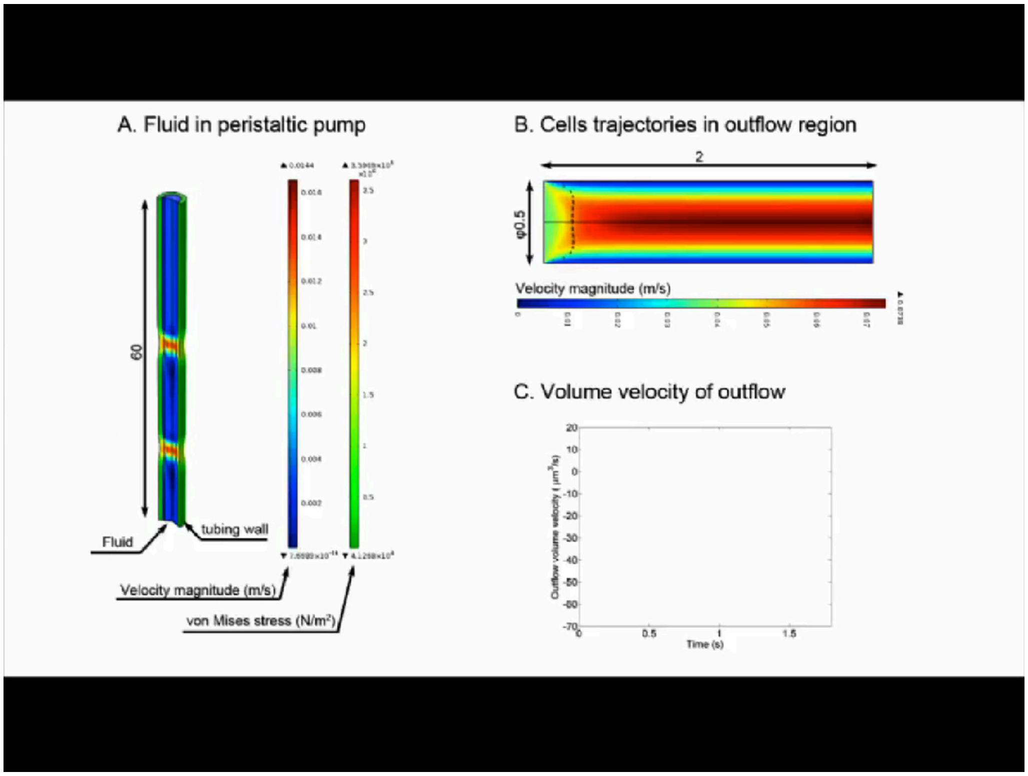

Supplementary Video S3: Simulation of the fluid SS generated by a microfluidic circulatory system. COMSOL Multiphysics 4.2a was used to analyze the flow oscillation in a peristaltic pump system. Two of the six metallic rollers were engaged at the same time to pump a viscous Newtonian fluid through an elastomeric tube. The model captured the flow oscillation caused by engaging and disengaging the rollers as well as the cell trajectories caused by this oscillation. **A.** The engaging and disengaging of the rollers caused flow fluctuations. The engaging speed of the roller was 75 bpm. The fluid viscosity was 0.01 dyne/cm^2. **B.** The flow fluctuations oscillated the cell trajectories. The cell concentration was 5×10^5/ml. **C.** The oscillation of the volume velocity with time was simulated with the same input in (A) and (B). Units are mm if not indicated.

See Supplementary File 3
